# Increased Th1 Cells with Disease Resolution of Active Pulmonary Tuberculosis in Non-Atopic Patients

**DOI:** 10.3390/biomedicines9070724

**Published:** 2021-06-24

**Authors:** Chun-Yu Lo, Yu-Chen Huang, Hung-Yu Huang, Fu-Tsai Chung, Chang-Wei Lin, Kian Fan Chung, Chun-Hua Wang

**Affiliations:** 1 Department of Thoracic Medicine, Chang Gung Memorial Hospital, Taipei 105, Taiwan; mixova.lo@gmail.com (C.-Y.L.); yuchenhahaha@gmail.com (Y.-C.H.); b9202071@cgmh.org.tw (H.-Y.H.); vikingchung@gmail.com (F.-T.C.); naturewei@adm.cgmh.org.tw (C.-W.L.); 2 College of Medicine, Chang Gung University, Taoyuan 333, Taiwan; 3 New Taipei Municipal TuCheng Hospital (Built and Operated by Chang Gung Medical Foundation), New Taipei City 236, Taiwan; 4 Airway Disease Section, National Heart and Lung Institute, Imperial College London and Royal Brompton Hospital, London SW3 6LY, UK; f.chung@imperial.ac.uk

**Keywords:** TB, chest radiograph score, atopy, Th1, Th2

## Abstract

Type 1 CD4^+^ T helper (Th1) cells mediate resistance to *Mycobacterium tuberculosis* (Mtb), and Th2 immunity generates specific immunoglobulin E upon allergen exposure. We investigated the impact of active tuberculosis (TB), atopic status, and anti-TB treatment on the balance between Th1 and Th2 (type 2 CD4^+^ T helper) immunity. CD4^+^/interferon (IFN)-γ^+^ Th1 cells (%Th1) and CD4^+^/interleukin-4^+^ Th2 cells (%Th2) in bronchoalveolar lavage (BAL) fluid and peripheral blood mononuclear cells (PBMCs) were measured by flow cytometry. The BAL %Th1 was higher in TB patients at baseline, compared to that in non-TB subjects, and was further increased in TB patients after stimulation with phorbol myristate acetate and ionomycin. The stimulated BAL %Th1 was inversely correlated with the severity score of chest radiography in TB patients. Heat-killed Mtb triggered more IFN-γ and nitrite production, as determined by enzyme-linked immunosorbent assay and the Griess reaction, respectively, from the alveolar macrophages of TB patients than that of non-TB subjects. Non-atopic TB participants had a higher %Th1 in PBMCs, compared to atopic individuals, and their %Th1 decreased after 3-month anti-TB treatment. Th1 response is provoked by active TB infection, is associated with less severe radiographic changes, is reduced in atopic patients with active TB infection, and is attenuated after anti-TB treatment.

## 1. Introduction

CD4^+^ T-helper (Th) cells orchestrate the immune system through the production of cytokines [1]. Upon exposure to intracellular microorganisms and allergens, macrophages and dendritic cells process these antigens and present them to naïve T cells, directing the differentiation of interferon (IFN)-γ^+^ type 1 CD4^+^ T helper (Th1) cells and interleukin (IL)-4^+^ type 2 CD4^+^ T helper (Th2) cells, respectively. These Th cells antagonize each other and are modulated by regulatory T cells. The imbalance of Th cell cytokines is likely to determine clinical outcomes.

Tuberculosis is an infection that affects 23% of the world’s population. Chest radiography is often used to screen for tuberculosis and to stratify the severity of the disease [2]. The rate of disease progression depends on the type of immune activation and inflammation that is generated. Th1 cells produce IFN-γ to activate alveolar macrophages (AMs), which helps to limit the spread of *Mycobacterium tuberculosis* (Mtb), and the release of nitric oxide and related reactive nitrogen intermediates helps in the clearance of mycobacteria [3,4,5]. AMs also secrete IL-12 and IL-23 to induce the generation of IFN-γ by Th1 cells and natural killer cells, increasing phagocytosis, phagolysosomal fusion, and oxidative burst [6]. Indeed, mice deficient in CD4^+^ subset and Th1 type cytokines (such as IL-12p40 and IFN-*γ*) succumb early to mycobacterial infection with high bacterial loads [7,8]. An earlier increase in antigen-specific effector Th1 cells restricts mycobacterial growth in the lung of Bacillus Calmette–Guérin-vaccinated mice [9]. Active pulmonary tuberculosis (TB) patients with the less extensive disease have a higher level of IFN-γ and IL-2 in their serum than those with more advanced disease [10]. Conversely, post-tuberculosis infection is characterized by consolidation, centrilobular nodules, and cavities (necrotizing granulomas), or the delayed resolution of chest radiographic changes, indicating a failing immune response [11].

Th2 cells and type 2 innate lymphoid cells release Th2 cytokines, including IL-4, IL-5, IL-9, and IL-13, leading to immunoglobulin (Ig) E production by B cells, eosinophilia, mast cell activation, secretion of mucus by goblet cells, and M2 macrophage polarization. These cytokines target helminths and facilitate tissue repair, and also contribute to atopic disorders, such as bronchial asthma, allergic rhinitis, and atopic dermatitis [12]. Atopy is an inherited predisposition toward the development of hypersensitivity in response to environmental allergens [13] but can also be present as an asymptomatic sensitization to allergens without the development of clinical allergy [14]. Defective lymphocyte regulation may also contribute to the development of atopic diseases [15]. Patients with multidrug-resistant TB have been shown to have downregulated expression of IFN-γ and IL-2 in the lungs, while on the other hand, upregulation of IL-4, IL-6, and tumor necrosis factor (TNF)-α expression during progressive disease [16]. Th2 cytokines inhibit autophagy in AMs and interfere with their ability to control intracellular *M. tuberculosis* [17]. The development of the Th2 response may efficiently antagonize protective cytokines, which may result in loss of TB control [18]. 

The aim of this study was to establish the role of Th1 and Th2 cells in peripheral blood and the lower respiratory tract in cellular immunity against mycobacterial invasion. In this regard, we hypothesized that the number of Th1 cells is increased during active TB infection, is associated with the extent of radiographic abnormalities, is attenuated in subjects with a concomitant atopic background, and is reduced after anti-TB treatment.

## 2. Materials and Methods

### 2.1. Subjects

In total, 19 patients with pulmonary TB, aged between 18 and 75 years, (12 men and 7 women with a mean age of 50.5 ± 3.7 years) were studied. In all patients, at least one recent sputum specimen was positive for acid-fast bacilli on microscopic examination using the auramine fluorochrome procedure and grew Mtb [19]. Sputum was collected on three consecutive days and the AFB smear was graded as follows: (0), absence of bacilli; (1), 1–9 bacilli; (2), 10–29 bacilli; (3), more than 30 bacilli per 30 oil immersion fields. The gradings over three days were summed as an index of bacterial sputum load [19]. A posteroanterior radiograph was taken for all patients at the time of enrolment. A grading of the extent of disease proposed by the World Health Organization (1960) was adopted [20]: 0 = no involvement; 1 = trivial, minimal lesions regarded as inactive; 2 = slight, minimal or rather larger lesion regarded as active; 3 = limited, lesions of greater extent, involving a total area of lung less than the right upper lobe; 4 = moderate, lesions of greater extent than in 3 but whose total extent, even if bilateral, not exceeding an area equivalent to the whole of one lung; 5 = extensive, lesions involved an area of more than the whole of one lung; 6 = gross, very extensive bilateral diseases. The radiographs were assessed by two independent chest physicians, and they arrived at an agreed grading for each patient. The control subjects consisted of seven nonsmoking, non-TB volunteers (3 men and 4 women, mean age of 45.7 ± 5.4 years) who were either healthy people or subjects asking for early lung cancer screening and agreed to receive bronchoscopy for bronchoalveolar lavage sampling. None of them had a history of lung disease based on physical and chest radiographic examinations. None of the TB or non-TB participants had upper respiratory tract infections within the last six weeks. None were taking antibiotics, corticosteroids, or other immunosuppressants at the time of evaluation. The study was approved by the Institutional Review Board of Chang Gung Memorial Hospital. The informed consent was obtained from each participant. 

### 2.2. Assessment of Atopic Status

Serum samples were separated from 10 mL of peripheral whole blood and assessed for automated immunoassay testing using ImmunoCAP (Phadia Laboratory Systems, Uppsala, Sweden) for total IgE (KU/L) and specific IgE antibodies to common allergens (i.e., house dust mite mix, grass mix, mold mix, and animal epithelia mix). Positive atopic status was defined as any positive ImmunoCAP test for allergen-specific IgE (>100 KU/L). Of those, all atopic patients showed elevated specific IgE to house dust allergen, e.g., *Dermatophagoides pteronyssinus, Dermatophagoides farinae*, and *Blattella germanica*. Their clinical features included four allergic rhinitis, one food allergy to crustacean shellfish (shrimp and crab), one asthma, and one atopic dermatitis. 

### 2.3. Preparation of Cells in Bronchoalveolar Lavage (BAL) Fluid 

BAL was performed in all study subjects using five aliquots (50 mL each) of 0.9% saline solution, as described previously [5,19]. Briefly, sterile saline solution was introduced into the involved bronchi of TB patients or into the right fourth or fifth subsegmental bronchus in non-TB subjects. The collected BAL fluid was centrifuged and the cell pellet was washed sequentially and resuspended in RPMI-1640 supplemented with 5% FCS at 10^6^ cells per milliliter. The cell viability was determined by trypan blue exclusion. Differential cell counts were determined by counting 500 cells on the cytocentrifuge preparations using modified Wright–Giemsa stain.

### 2.4. Quantification of Cytokines and Nitrite Produced by Cultured BAL Cells

BAL cells were placed in plastic culture dishes in RPMI-1640 for 90 min, washed to remove nonadherent cells, and scraped off with a sterile rubber policeman. To investigate whether the synergistic effect of mycobacterial antigen and IFN-γ activated by Th1 cells can enhance NO production from alveolar macrophages and inhibit Th2 immune response, isolated BAL cells were cultured at 10^6^ cells/mL in 12-well Petri dishes for 24 h in RPMI-1640 medium containing 5% FCS, penicillin 100 U/mL, and streptomycin 100 μg/mL, in the presence or absence of 5 μg/mL heat-killed Mtb H37Ra (Difco Laboratories, Detroit, Mich) [5]. The culture supernatant was collected and frozen at −70 °C before the measurement of cytokines and nitrite. IFN-γ and IL-4 were determined by enzyme-linked immunosorbent assay. Nitrite quantification of the culture medium was measured using the Griess reaction [5]; a total of 100 μL of culture medium aliquots was mixed with equal volumes of Griess reagent (100 μL: 1% sulphanilamide and 0.1% naphthyl-ethylenediamide in 5% phosphoric acid; Invitrogen) and incubated at room temperature for 10 min. The absorbance at 550 nm was measured in a microplate reader (Biotek, Winooski, VT, USA). Sodium nitrite was used as the standard.

### 2.5. Preparation of Peripheral Blood Mononuclear Cells (PBMCs)

For this procedure, 30 milliliters of heparinized blood was collected from the TB and non-TB participants. PBMCs were isolated on a Ficoll-Paque^TM^ PLUS (GE HealthCare, Uppsala, Sweden) density gradient centrifugation and were then resuspended (10^6^ cells/mL) in RPMI 1640 (GIBCO, Grand Island, NY, USA) medium containing 5% fetal calf serum (FCS; Flow Laboratories, Paisley, Scotland, UK), 100 U/mL penicillin, and 100 g/mL streptomycin. 

### 2.6. Identification of Th1 and Th2 Cells in Peripheral Blood and BAL Fluid

**Figure 1:**
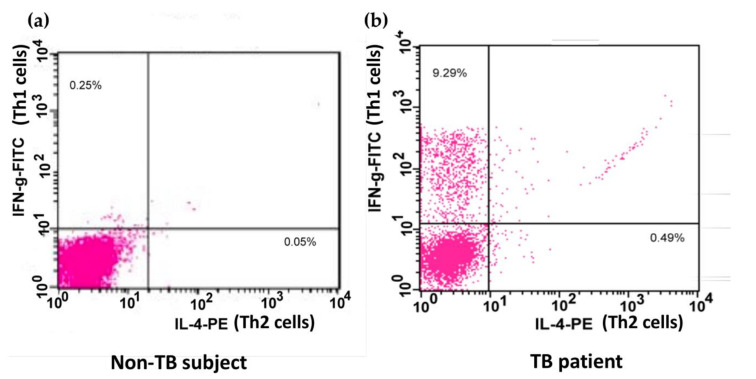
Flow cytometric analysis of Th cells. A demonstration of the expression of IL-4 and IFN-γ on phorbol myristate acetate and ionomycin-stimulated peripheral blood CD4^+^ Th cells from one non-TB subject (**a**) and one patient infected with TB (**b**). Peripheral blood mononuclear cells were stained with PerCP-conjugated anti-Leu-3a/CD4 antibody, FITC-conjugated anti-INF-γ antibody, and PE-conjugated anti-IL-4 antibody or the respective isotype IgG controls. The expression of IFN-γ and IL-4 on Th cells was identified by flow cytometry. Th, CD4^+^ T helper; TB, tuberculosis; PerCP, peridinin–chlorophyll protein; FITC, fluorescein isothiocyanate; PE, phycoerythrin; IL-4, interleukin-4; IFN-γ, interferon-γ.

The application of procedures to explore Th1 and Th2 cells was modified as described previously [21]. Unstimulated PBMCs and BAL cells (1 × 10^6^ cells/mL) or cells stimulated with phorbol myristate acetate (PMA; 1 ng/mL, Sigma-Aldrich, Poole, UK) and ionomycin (IONO; 500 ng/mL, Sigma) in vitro were stained with monoclonal antibodies of anti-Leu-3a/CD4 peridinin–chlorophyll protein (PerCP; Becton Dickinson, San Jose, CA, USA) or IgG isotype controls for 30 min at 0 °C. Cells were washed twice using phosphate-buffered saline (PBS; Sigma-Aldrich, Poole, UK), mixed at 5 × 10^5^ cells/mL before using PBS twice, mixed with 500 L of FACS^TM^ permeabilizing solution (1×, Becton Dickinson, San Jose, CA, USA) at room temperature for 10 min and then washed twice extensively. The cells were then incubated in the presence of monoclonal antibodies of anti-INF-γ fluorescein isothiocyanate (FITC; Becton Dickinson)/anti-IL-4 phycoerythrin (PE; Becton Dickinson) or the corresponding isotype controls in the dark for 1 h at 4 °C. The cells were then washed and proceeded to flow cytometric analysis with a FACScan flow cytometer (Beckon Dickinson, Mountain View, CA, USA) and LYSYS II software (Beckon Dickinson, Mountain View, CA, USA). The expression of CD4^+^/interferon-γ^+^ Th1 and CD4^+^/interleukin-4^+^ Th2 in the BAL cells or PBMCs was analyzed by flow cytometry (Figure 1).

### 2.7. Statistical Analysis

Data are presented as the mean ± standard error of the mean (SEM). Statistical analysis was carried out using the GraphPad Prism v.7 software package (GraphPad Prism Software Inc., San Diego, CA, USA). The Wilcoxon matched-pairs test was used to compare two related samples (before and after PMA+IONO stimulation, before and after anti-TB treatment, etc.). The differences between two disease groups (e.g., TB and non-TB, or atopic and non-atopic) were determined by the Mann–Whitney test or chi-square test. The differences amongst ≥three disease groups (different scores in the chest radiography) were determined by the Kruskal–Wallis test, followed by Dunn’s post hoc test. Correlations were determined by Spearman’s rank correlation. A *p*-value < 0.05 was considered statistically significant.

## 3. Results

Nineteen TB-infected patients (including seven atopic subjects) and seven non-TB control subjects had received bronchoscopy within three days after the initiation of anti-TB treatment. Results indicate that 1 of 12 non-atopic TB patients (8.3%) and 3 of 7 atopic TB patients (42.9%) remained positive for tuberculosis culture after 28 days of anti-TB treatment (chi-square 3.17, *p* = 0.075). All subjects achieved sputum conversion at the 56-day follow-up. The cellularity of BAL cells and the proportion of AMs, lymphocytes, and neutrophils were significantly different between subjects with and without TB (Table 1). Among the TB patients, the cellularity of the BAL fluid was significantly higher in the non-atopic TB patients, compared to that of the atopic TB patients. Both the non-atopic and atopic TB patients had a higher proportion of lymphocytes and neutrophils in the BAL fluid than those of the control subjects. However, the proportion of neutrophils in BAL was significantly increased in the non-atopic TB patients, compared with the atopic TB patients. There was no difference in the proportion of eosinophils in the BAL fluid between TB patients with or without atopy and the control subjects (Table 1). 

AMs were isolated from BAL fluid by the adherence method and cultured in 1 mL of the medium at the concentration of 10^6^ cells/mL for 24 h, and supernatants were then collected for analysis [5]. The AMs from the TB patients had greater IFN-γ production (377 ± 116 ng/mL) at baseline, compared with non-TB subjects (48 ± 32 ng/mL; *p* = 0.022), and further increased upon stimulation with 5 μg/mL heat-killed Mtb H37Ra (4649 ± 1512; *p* = 0.025, compared with unstimulated, and *p* = 0.001, compared with the non-TB subjects; Table 2). The heat-killed Mtb-stimulated AMs from the TB patients also generated more nitrite (4669 ± 610 μM), compared with the non-TB subjects (1731 ± 184 μM; *p* = 0.001). The release of IL-4 was below the limit of detection of the assay in all specimens.

**Figure 2:**
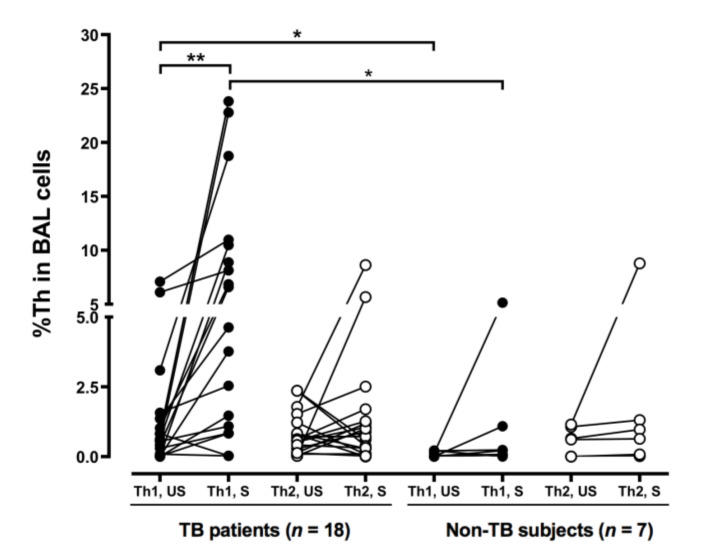
More BAL Th1 cells in the patients with tuberculosis. Cells from the TB patients and the non-TB subjects were collected by BAL and stimulated with phorbol myristate acetate and ionomycin. CD4^+^/IFN-γ^+^ Th1 and CD4^+^/IL-4^+^ Th2 cells were identified by flow cytometry. The difference between cell profiles pre- and post-stimulation was determined by a paired *t*-test. The difference between cell profiles in participants with and without TB was determined by the Mann–Whitney test. * *p* < 0.05 and ** *p* < 0.01. BAL, bronchoalveolar lavage; Th1, type 1 CD4^+^ T helper; Th2, type 2 CD4^+^ T helper; %, percentage of CD4^+^ T helper cells; TB, tuberculosis; IL-4, interleukin-4; IFN-γ, interferon-γ; US, unstimulated; S, stimulated.

The baseline proportion of IFN-γ-expressing CD4^+^ cells (unstimulated %Th1) was higher in the TB patients, compared to that in the non-TB subjects (*p* < 0.05) (Figure 2). These cells were also stimulated with PMA and IONO in vitro [21]. PMA is an analog of diacylglycerol that diffuses through the cell membrane into the cytoplasm, where it directly activates protein kinase C (PKC). IONO, a calcium ionophore, is also used to trigger calcium release from the endoplasmic reticulum, activating calcium-sensitive enzymes and synergizing with PMA. The combination of PMA and IONO resulted in strong signaling and subsequent cytokine production used for the identification of subsets of CD4^+^ cells. Indeed, the %Th1 was further increased (*p* < 0.05) in the TB patients after incubation in the presence of PMA and IONO, compared to that in the non-TB subjects (*p* < 0.01) (Figure 2). The proportion of IL-4-expressing CD4^+^ cells (%Th2) in the BAL fluid did not differ between the subjects with and without TB and was not affected by PMA and IONO stimulation.

**Figure 3:**
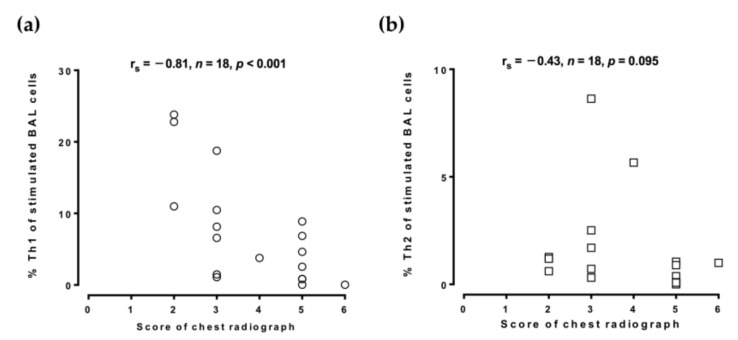
Inverse association between the proportion of BAL Th1 cells and chest radiographic severity score. Cells from the TB-infected patients and non-TB subjects were collected by bronchoalveolar lavage and stimulated with phorbol myristate acetate and ionomycin. CD4^+^/IFN-γ^+^ Th1 cells and CD4^+^/IL-4^+^ Th2 cells were identified by flow cytometry. (**a**,**b**) The association between the chest radiographic severity score and the proportion of Th1 cells or Th2 cells was determined by Spearman’s rank correlation. BAL, bronchoalveolar lavage; %Th1, percentage of type 1 CD4^+^ T helper cells; %Th2, percentage of type 2 CD4^+^ T helper cells; TB, tuberculosis; IL-4, interleukin-4; IFN-γ, interferon-γ.

To evaluate the role of Th1 and Th2 cells in disease progression, the chest radiographic films were reviewed by two chest physicians. The score of chest radiographic severity in the TB patients was inversely correlated with stimulated BAL %Th1 (r_s_ = −0.81, *p* < 0.001; Figure 3a) but was not correlated with stimulated BAL %Th2 (r_s_ = −0.43, *p* = 0.095; Figure 3b), although the score of chest radiographic severity and the unstimulated BAL %Th1 (r = −0.23, *p* = 0.354) or the unstimulated BAL %Th2 (r = −0.32, *p* = 0.20) was not correlated significantly (not shown in the figures).

**Figure 4:**
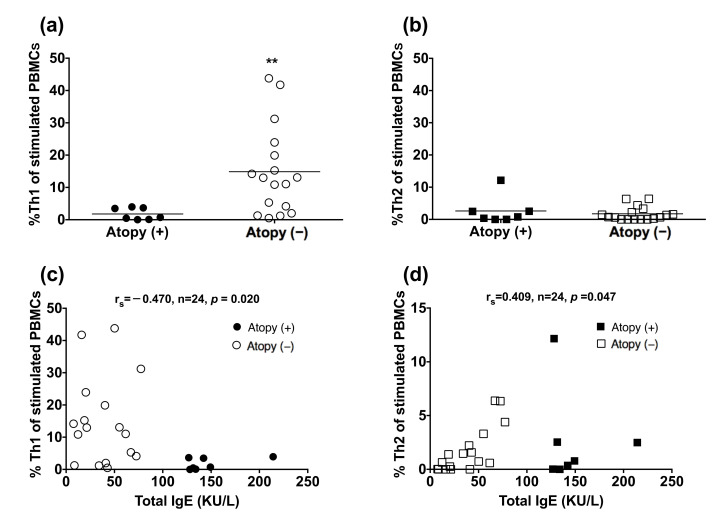
Reduced peripheral blood Th1 cells in the atopic patients. Peripheral blood mononuclear cells from TB-infected patients were stimulated with phorbol myristate acetate and ionomycin. CD4^+^/IFN-γ^+^ Th1 cells and CD4^+^/IL-4^+^ Th2 cells were identified by flow cytometry: (**a**,**b**) values are shown as individual data points and medians. The difference between cell profiles in the atopic and non-atopic patients was determined by a paired *t*-test; (**c**,**d**) The association between the total IgE level and the proportion of Th1 cells or Th2 cells was determined by Spearman’s rank correlation. ** *p* < 0.01. %Th1, percentage of type 1 CD4^+^ T helper cells; %Th2, percentage of type 2 CD4^+^ T helper cells; Ig, immunoglobulin; TB, tuberculosis; IL-4, interleukin-4; IFN-γ, interferon-γ.

Since the balance between Th1 and Th2 responses could be skewed by infection and allergy, we analyzed the effect of atopy on %Th1 and %Th2 in TB subjects. Peripheral blood mononuclear cells (PBMCs) were isolated from patients with TB. The non-atopic subjects had a higher blood %Th1 in CD4^+^ cells upon PMA and IONO stimulation, compared to the atopic subjects (*p* = 0.0035; Figure 4), but blood %Th2 was not significantly different between the two groups. Total IgE level was inversely correlated with blood %Th1 (rs = −0.470, *p* = 0.020) and positively correlated with blood %Th2 (rs = 0.409, *p* = 0.047).

**Figure 5:**
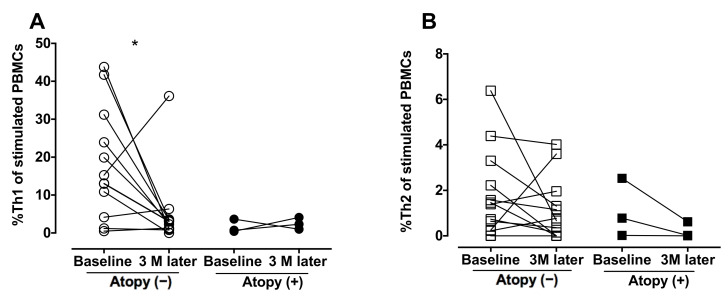
Decrease in peripheral blood Th1 cells after anti-TB treatment. Peripheral blood mononuclear cells were obtained from the TB-infected patients before and after the 3-month anti-TB treatment and stimulated with phorbol myristate acetate and ionomycin. CD4^+^/IFN-γ^+^ Th1 cells and CD4^+^/IL-4^+^ Th2 cells were identified by flow cytometry. (**A**,**B**) The difference between cell profiles before and after medication was determined by paired a *t*-test. * p < 0.05. %Th1, percentage of type 1 CD4^+^ T helper cells; %Th2, percentage of type 2 CD4^+^ T helper cells; TB, tuberculosis; IL-4, interleukin-4; IFN-γ, interferon-γ; PBMCs, peripheral blood mononuclear cells.

The analysis of blood CD4 cells was repeated 3 months after the initiation of anti-TB medication (Figure 5). Treatment resulted in a reduction in %Th1 in the non-atopic individuals (*p* = 0.031) but did not influence %Th2 in the atopic TB patients or %Th2 in the TB patients with or without atopy. 

## 4. Discussion

Host immunity against Mtb infection has often been described as being orchestrated by Th1 cells [4]. We confirmed the dominant role of Th1 cells in the lower respiratory tract in active tuberculosis, and its frequency was correlated inversely with the severity of the radiographic abnormality. The blood %Th1 was only increased in the non-atopic TB-infected patients, and the increase was less significant after three months of anti-TB chemotherapeutic treatment.

The high BAL %Th1 reflects the robust IFN-γ responses at the site of disease. In patients with tuberculous pleuritis, the increase in IFN-γ mRNA and the decrease in IL-4 mRNA were even more significant in the pleural fluid cells, compared with those from PBMCs [22]. The concentration of IFN-γ in the supernatants of Mtb-stimulated pleural fluid cells was higher than the corresponding concentrations in the supernatants of stimulated PBMCs [22]. 

Although Mtb may reduce nitrate (NO_3_^−^) to nitrite (NO_2_^−^) as a means to maintain redox homeostasis and energy production under hypoxic conditions [23], the mycobactericidal effect of IFN-γ- and TNF-α-activated murine AMs also correlates with the induction of the L-arginine-dependent generation of nitric oxide (NO), nitrogen dioxide (NO_2_), and nitrous acid (HNO_2_) [24]. These toxic reactive nitrogen intermediates are responsible for killing and inhibiting the growth of virulent Mtb. Heat-killed Mtb has been shown to increase NO production and inducible nitric oxide synthase (iNOS) expression by the AMs of rats upon IFN-γ stimulation in vivo and to cause a greater release of IFN-γ and nitrite from the AMs of active TB patients than in the AMs of non-TB subjects. The synergistic effect of Mtb antigen and IFN-γ induces NO production and inhibits Th2 immune response, playing a crucial role in the suppression of intracellular mycobacterium [25]. In addition, the Th2 cytokine IL-13 reduces iNOS expression [26] and blocks PMA-induced NO production in human monocytes [27]. Taken together, the upregulation in IFN-γ and NO produced by activated Th1 cells contributes to local mycobactericidal cellular immunity in pulmonary TB.

We also demonstrated an inverse association between the number of Th1 cells in the BAL fluid and the radiographic abnormality amongst the tuberculosis-infected subjects. It has been reported that a mutation in the gene for the IFN-γ receptor leads to a functional defect in the upregulation of TNF-α by AMs, resulting in severe mycobacterial infection in children [28]. Mice unable to produce IFN-γ upon Mtb infection have been shown to exhibit heightened tissue necrosis and to succumb to a rapid and fatal course of TB that could be delayed by treatment with exogenous IFN-γ [29]. Lower BAL levels of IL-6 and IFN-γ-inducible protein-10 (IP-10) have been associated with the development of cavitation in TB-infected patients [30]. Compared to the radiologically normal lobes in the same individuals, a lower baseline number of IFN-γ-expressing CD4^+^ cells can be observed in the BAL fluid recovered from the sites of tuberculous cavitation, with a weaker IFN-γ production in response to tuberculin stimulation [31], suggesting that the composition of Th1 cells in BAL may provide valuable information on the progression of TB.

The lack of shift of Th1/Th2 balance toward Th1 response could be associated with atopy. Th2 cells release IL-4, promoting IgE production by B cells and counter-regulating T-bet-mediated Th1 immune response, including the induction of IFN-γ [32]. IL-4 reduces mycobacterial containment in infected AMs, leading to decreased levels of INF-γ and TNF-α in CD4^+^ T cells and an increase in regulatory T cells [33]. A higher IgE level has been found in patients with cavitary and radiologically disseminated TB [34]. There is an inverse association between tuberculin-induced delayed hypersensitivity and childhood atopic disorders, including asthma in Japan and atopic dermatitis and allergic rhinitis in Taiwan [35,36]. The present study extended these observations to active TB infection, in which there was a significant reduction in blood Th1 cells in the atopic patients. Another common situation associated with the Th2-skewed immune response is a parasitic infestation. A diminution in mycobacterial-specific Th1 cells and Th17 cells and their relevant cytokines (i.e., IFN-γ, TNF-α and IL-2, IL-17_A_, and IL-17_F_) has been found in patients with concomitant filarial or Strongyloides infection [37]. The production of IFN-γ by tuberculin-stimulated mononuclear cells has been shown to be reduced in filarial patients, which can be restored by diethylcarbamazine-mediated parasitic clearance [38].

A decline in peripheral blood Th1 cells was observed after three months of anti-TB treatment in our non-atopic Mtb-infected patients. The change in the %Th1 in the atopic patients and the change of %Th2 in both the atopic and non-atopic patients was not remarkable, possibly due to the relatively low baseline levels in these participants. A negative conversion of the interferon-γ-release assay was found in 34.6% of TB contacts six months after the initiation of daily isoniazid and rifampicin chemoprophylaxis for three months in a South Korean cohort [39]. Intriguingly, the study also demonstrated a transient increment in IFN-γ level at three months before it dropped after a medication-free three-month period, which might reflect the restoration of cellular immunity against Mtb [40]. We also found that the proportion of sputum culture positive for Mtb at 28 days was higher in non-atopic TB patients, compared to atopic subjects. It is evident that the Th2 phenotype of atopic subjects can suppress the Th1 immunity. When pulmonary TB is effectively treated in atopic subjects, fatal asthma [41] or uncontrolled atopic dermatitis may develop. Despite good asthma control at the initial diagnosis of TB infection, some asthma patients experienced poorly controlled asthma during the anti-TB treatment, followed by gradual recovery [41]. In this situation, downregulated Th1 immunity with a subsequent Th2 flare-up should be taken into consideration in order to manage the suppression of the Th2 immune response effectively. Serial evaluation of the dynamic change in Th1 cells during anti-TB treatment is warranted.

The results of our study may have been influenced by the limitations of the patient population and study design. First, the difference between two groups after stimulation might not be present in vivo since Th1 response by BAL cells of non-atopic Tb patients was not statistically different from atopic TB patients. Second, earlier chest images were not always available in all participants, and therefore, it was difficult to distinguish post-primary reactivation from repeated infection of a previously sensitized host or even primary TB. However, these patients could have different cell profiles. Third, the small sample size of the enrolled subjects may have been underestimated to detect the shifts in the Th1/Th2 balance during Mtb infection in the atopic patients. On the basis of our present pilot study, it is worth recruiting more participants for longitudinal analysis of the impact of atopy or allergic diseases (such as asthma) on the therapeutic outcome of anti-TB treatment and Th1/Th2 polarization in TB patients. In addition, we would like to conduct an in vivo study using mycobacterium-infected Th2-biased ovalbumin-challenged or GATA-3 transgenic animals to better dissect the interaction between allergy and Mtb infection in the future.

In conclusion, Th1 response is provoked by active TB infection and is associated with less radiographic severity, a lower response in atopic subjects, and attenuation after anti-TB treatment. When atopic symptoms deteriorate during the process of anti-TB treatment, this could be a sign of exaggerated Th2 immunity resulting from a suppressed Th1 response. Our findings highlight the role of BAL in the evaluation of TB patients, and this research model should be applied to the investigation of the role of regulatory T cells and Th17 cells in pulmonary TB. 

## Figures and Tables

**Table 1:** Characteristics of bronchoalveolar lavage cells in control subjects and patients with active pulmonary TB.

KERRYPNX	Control Subjects (*n* = 7)	TB Patients		
		Total (*n* = 19)	Non-Atopy (*n* = 12)	Atopy (*n* = 7)
Age, years	45.7 ± 5.4	50.5 ± 3.7	52.8 ± 5.0	46.4 ± 5.6
Male/female	3/4	12/7	8/4	4/3
IgE, IU/mL	56.1 ± 15.4	78.1 ± 13.5	38.1 ± 5.6 ^¶^	146.6 ± 11.7 **
Bacterial load on sputum	N/A	3.5 ± 1.8	4.6 ± 1.1	1.7 ± 1.0
Bronchoalveolar lavage				
Cellularity, 10^5^ cells/mL	1.6 ± 0.2	10.6 ± 3.0 **	12.9 ± 4.5 ^¶,^**	6.6 ± 2.7
Recovery rate, %	50.1 ± 7.1	44.4 ± 3.1	43.2 ± 4.1	46.5 ± 5.1
Viability, %	95.4 ± 1.3	91.1 ±1.5	92.3 ± 1.8	88.9 ± 2.7
AMs, %	97.1 ± 0.6	83.8 ± 2.3 **	80.3 ± 4.0 **	88.6 ± 2.3 *
Lymphocytes, %	1.7 ± 0.4	7.3 ± 1.3 *	6.8 ± 1.6 *	8.2 ± 2.3 *
Neutrophils, %	0.8 ± 0.3	9.8 ± 2.9 **	14.2 ± 4.2 ^§,^**	2.2 ± 1.0 *
Eosinophils, %	0.3 ± 0.3	0.8 ± 0.2	0.8 ± 0.3	0.9 ± 0.4

Values presented as mean ± standard error of the mean (SEM). * *p* < 0.05 and ** *p* < 0.01 compared with the control subjects. ^§^ *p* < 0.05 and ^¶^ *p* < 0.01 compared with the atopic TB patients. The recovery rate was the total recovered volume divided by the 250 mL instilled volume. AMs, alveolar macrophages; TB, tuberculosis; Ig, immunoglobulin; N/A, not available.

**Table 2:** IFN-γ and nitrite levels from the alveolar macrophages of patients with active TB and non-TB subjects.

	Control Subjects (*n* = 5)		TB Patients (*n* = 9)	
	Unstimulated	Stimulated	Unstimulated	Stimulated
IFN-γ (ng/mL)	48 ± 32	26 ± 13	377 ± 116 ^¶¶^	4649 ± 1512 *^,¶¶¶^
Nitrite (μM)	2995 ± 918	1731 ± 184	3036 ± 336	4669 ± 610 *^,¶¶¶^
IL-4 (ng/mL)	Undetectable	Undetectable	Undetectable	Undetectable

Alveolar macrophages from active TB patients and non-TB subjects were cultured at 10^6^ cells/mL, and the level of interferon-γ, nitrite, and interleukin-4 were determined. * *p* < 0.05 compared to the unstimulated specimens; ^¶¶^ *p* < 0.01 an ^¶¶¶^ *p* < 0.001 compared with the control subjects. TB, tuberculosis; IFN-γ, interferon-γ; IL-4, interleukin-4.

## Data Availability

The data sets analyzed during the current study are available from the corresponding author upon reasonable request.
